# Geospatial and age-related patterns of *Taenia solium* taeniasis in the rural health zone of Kimpese, Democratic Republic of Congo

**DOI:** 10.1016/j.actatropica.2016.03.013

**Published:** 2017-01

**Authors:** Joule Madinga, Kirezi Kanobana, Philippe Lukanu, Emmanuel Abatih, Sylvain Baloji, Sylvie Linsuke, Nicolas Praet, Serge Kapinga, Katja Polman, Pascal Lutumba, Niko Speybroeck, Pierre Dorny, Wendy Harrison, Sarah Gabriel

**Affiliations:** a Institute of Health and Society, Université Catholique de Louvain, Brussels, Belgium; b Department of Biomedical Sciences, Institute of Tropical Medicine, Antwerp, Belgium; c Institut National de Recherche Biomédicale, Kinshasa, Democratic Republic of the Congo; d Zone de santé Rurale de Kimpese, Democratic Republic of the Congo; e Programme National de Lutte contre la Trypanosomiase Humaine Africaine, Kinshasa, Democratic Republic of the Congo; f Department of Tropical Medicine, University of Kinshasa, Democratic Republic of the Congo; g Department of Infectious Disease and Epidemiology, Imperial College, London, UK

**Keywords:** Taeniasis, Porcine cysticercosis, Epidemiology, Spatial clustering, Democratic republic of Congo

## Abstract

**Background:**

*Taenia solium* infections are mostly endemic in less developed countries where poor hygiene conditions and free-range pig management favor their transmission. Knowledge on patterns of infections in both human and pig is crucial to design effective control strategies. The aim of this study was to assess the prevalence, risk factors and spatial distribution of taeniasis in a rural area of the Democratic Republic of Congo (DRC), in the prospect of upcoming control activities.

**Methods:**

A cross-sectional study was conducted in 24 villages of the health zone of Kimpese, Bas Congo Province. Individual and household characteristics, including geographical coordinates were recorded. Stool samples were collected from willing participants and analyzed using the copro-antigen enzyme-linked immunosorbent assay (copro-Ag ELISA) for the detection of taeniasis. Blood samples were collected from pigs and analyzed using the B158/B60 monoclonal antibody-based antigen ELISA (sero-Ag ELISA) to detect porcine cysticercosis. Logistic regression and multilevel analysis were applied to identify risk factors. Global clustering and spatial correlation of taeniasis and porcine cysticercosis were assessed using K functions. Local clusters of both infections were identified using the Kulldorff’s scan statistic.

**Results:**

A total of 4751 participants above 5 years of age (median: 23 years; IQR: 11–41) were included. The overall proportion of taeniasis positivity was 23.4% (95% CI: 22.2–24.6), ranging from 1 to 60% between villages, with a significant between-household variance of 2.43 (SE = 0.29, p < 0.05). Taeniasis was significantly associated with age (p < 0.05) and the highest positivity was found in the 5–10 years age group (27.0% (95% CI: 24.4–29.7)). Overall, 45.6% (95% CI: 40.2–51) of sampled pigs were sero-positive. The K functions revealed a significant overall clustering of human and pig infections but no spatial dependence between them. Two significant clusters of taeniasis (p<0.001; n = 276 and n = 9) and one cluster of porcine cysticercosis (p<0.001; n = 24) were found.

**Conclusion:**

This study confirms high endemicity and geographical dispersal of taeniasis in the study area. The role of age in taeniasis patterns and significant spatial clusters of both taeniasis and porcine cysticercosis were evidenced, though no spatial correlation was found between human and pig infections. Urgent control activities are needed for this endemic area.

## Highlights

• We assessed patterns of taeniasis in 24 village communities of Kimpese health zone.
• Prevalence of taeniasis was very high and varied between households and villages.
• Children of five to ten years were the most infected age group.
• Taeniasis was not spatially correlated with porcine cysticercosis.

## Graphical abstract

**Figure:**
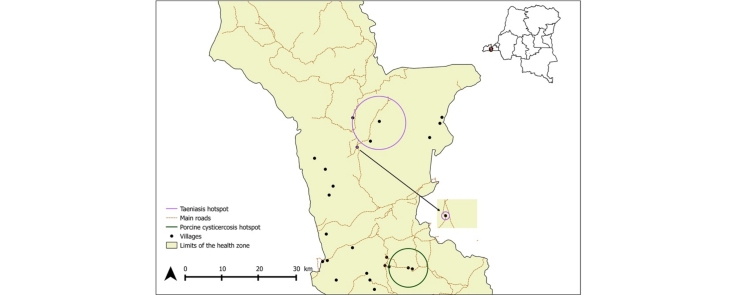

## 1 Introduction

*Taenia solium* is a cestode parasite, infecting both human and pigs and prevailing mostly in developing countries (Sciutto et al., 2000). The adult tapeworm develops in the intestine of the human host after ingestion of undercooked infected pork, causing taeniasis. Infective eggs are released via the stool of tapeworm carrier and contaminate the environment. Ingestion of these eggs by coprophagic pigs or by human through fecal-oral contamination, leads to establishment of the metacestode larval stage of the parasite (cysticerci) in hosts tissues, causing porcine and human cysticercosis, respectively. In human, the most dangerous location of cysts is the central nervous system, since neurocysticercosis can lead to epilepsy, epileptic seizures and severe neurological symptoms (Garcia et al., 2003b). Neurocysticercosis is the major cause of acquired epilepsy and is responsible for about 30% of seizures in endemic areas (Ndimubanzi et al., 2010). In addition, porcine cysticercosis is a source of economic losses due to confiscation of contaminated pork (Fan and Chung, 1997, Gonzalez et al., 2001) or significant reduction of its market value (Carabin et al., 2006, Praet et al., 2009).

Control of the complex taeniasis/cysticercosis can be achieved through different approaches, including mass treatment of adult *T. solium* tapeworm carriers (Garcia et al., 2007, Pawlowski, 2006, WHO, 2012). Mass chemotherapy has been used as a control strategy for taeniasis/cysticercosis using the anthelmintic niclosamide at 2 g (Allan et al., 1997) or praziquantel at 5–10 mg/kg (Sarti et al., 2000). Due to lack of adequate field-applicable diagnostic tools for taeniasis (Praet et al., 2013), the micro-geographical distribution of cysticercosis infected pigs has been suggested to be used as an indicator of the distribution of taeniasis infected human subjects. Clustering of porcine cysticercosis in specific households would then indicate the occurrence of tapeworm carriers in the vicinity, pointing to targeted screening and treatment, whereas a dispersed distribution would suggest a wider geographic spread of taenia carriers, pointing to the need for mass treatment.

In the Democratic Republic of Congo (DRC), the Ministry of Health has adopted, but not yet implemented a national plan against neglected tropical diseases (NTDs), including mass drug administration (MDA) against helminthiasis according to WHO guidelines (http://www.nyankunde.org/documentation/doc_22.pdf). However, data on most of those NTDs in DRC are still scarce (Rimoin and Hotez, 2013). Specifically for taeniasis/cysticercosis, a study recently conducted in 5 villages of the rural health zone of Kimpese in the west of the DRC, reported a 5.2% and a 41.2% porcine cysticercosis prevalence by lingual examination and circulating antigen detection respectively (Praet et al., 2010). Another study conducted in one of these villages reported a 21.6% prevalence of active human cysticercosis by circulating antigen detection with a 12.7‰ adjusted prevalence of active epilepsy and a 0.3% prevalence of taeniasis by coprology (Kanobana et al., 2011). These data suggest that *T. solium* infections may be (highly) endemic in this area. The current study aims to assess the prevalence, risk factors and spatial distribution of taeniasis in order to contribute in designing control strategies tailored to this setting.

## 2 Methods

### 2.1 Study area and population

**Fig. 1:**
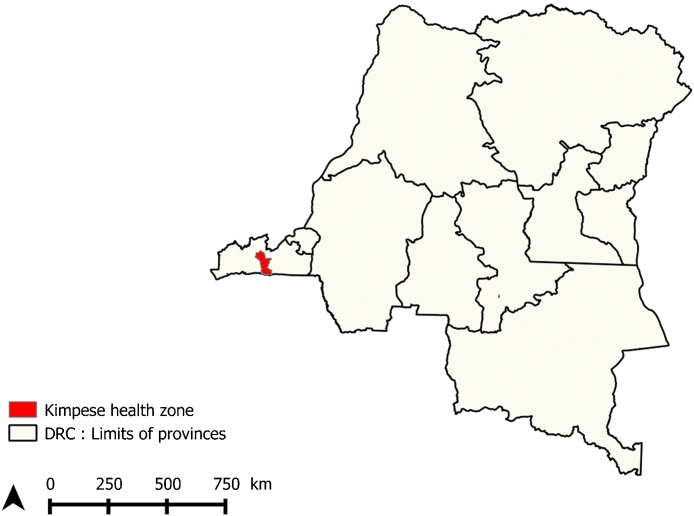
Map of the DRC showing the location of Kimpese health zone.

The study was conducted in the rural health zone of Kimpese, in the Bas-Congo Province (Fig. 1). In DRC, a health zone is the operational unit of the health system, in charge of implementation of primary health care strategies developed at the central level. Each health zone comprises health areas, which include a number of villages depending on one health center for primary health care (http://www.who.int/medicines/areas/coordination/drc_pharmaceutical_profile.pdf). Early 2011, the population of Kimpese health zone comprised around 150,482 inhabitants distributed over 20 health areas, including 519 villages (P. Lukanu, personal data). Agriculture represents the most important source of income in this area where pigs, goats and chickens are the most reared animals by farmers. Previous studies in this health zone reported a high number of free roaming pigs and occurrence of both human and porcine cysticercosis (Kanobana et al., 2011, Praet et al., 2010). Villages share cultural, commercial, social and economic characteristics. There is no piped water, roads are not paved and there is no electricity. Poor hygiene is widespread in the Bas-Congo province as only 26.8% of the population use toilets (http://www.afdb.org/fileadmin/uploads/afdb/Documents/Project-and-Operations/DRC).

#### 2.1.1 Study design and data collection

This study used cross-sectional baseline data from a community-based interventional study assessing the impact of MDA using praziquantel at 40 mg/kg (the dose used against schistosomiasis) on the prevalence of taeniasis and porcine cysticercosis. It is part of a multi-country project aiming to assess the safety and impact of MDA-based control in areas co-endemic for taeniasis/cysticercosis and schistosomiasis (Bill and Melinda Gates Foundation funded “Integrated control of taeniasis and cysticercosis” coordinated by Imperial College, London). The baseline survey took place between November 2011 and November 2012. Briefly, 24 villages in a radius of 50 km around Kimpese city were selected based on pre-determined inclusion criteria. These criteria included the presence of commonly known taeniasis/cysticercosis risk factors such as free roaming pigs, insufficient number and use of latrines; and the absence of other control initiatives such as sanitation programmes. All households of included villages were visited and all household members were invited to participate in the study, except children younger than 5 years, pregnant women, people with a history of epilepsy or seizures and people who had received a praziquantel treatment in the past 2 months. The head of each household was interviewed about the presence of household level risk factors of taeniasis/cysticercosis infection (e.g. pig breeding and presence of a latrine in the household). Geographic coordinates of each participating household were recorded using a Global Positioning System (GPS) receiver (eTrex LegendH Cx, Garmin). Another form was used to record individual data (age, gender, toilet use) from each participant.

#### 2.1.2 Sample collection and storage

Upon written inform consent, each participant was given one plastic sample bottle and requested to deliver a stool sample. Submitted stool samples were transported to the laboratory of Kimpese health zone where they were divided into two aliquots; one placed in 10% formalin and the other in 70% ethanol. The formalin aliquots were kept at room temperature while ethanol aliquots were kept at −20 °C for future molecular analysis.

Blood samples were collected from the cranial vena cava of each pig older than 3 months, after written informed consent from pig holders. The blood samples were placed in a cooler box immediately after collection and transported to the laboratory of the Health Zone of Kimpese where they were allowed to clot overnight at 4 °C. Then, the blood was centrifuged and the serum dispensed into 2 ml aliquots and stored in labeled cryogenic vials at −20 °C. All samples were shipped to the Regional Reference Laboratory for Cysticercosis in the School of Veterinary Medicine, University of Zambia, Lusaka, Zambia for subsequent analyses.

#### 2.1.3 Laboratory analysis

Human stool samples were tested for the presence of taenia antigen, by an in-house copro-antigen detection ELISA (copro-Ag ELISA) as described by Allan et al. (Allan et al., 1990), with slight modifications, as described by Mwape et al. (Mwape et al., 2012). The test results were obtained by comparing the optical density (OD) of each stool sample with the mean of a series of 8 reference negative stool samples from DRC plus 3 standard deviations (cut off).

Pig sera were submitted to the monoclonal antibody based B158/B60 Ag-ELISA (sero-Ag ELISA) (Brandt et al., 1992, Dorny et al., 2004a) to detect the presence of circulating cysticercus antigens. The positive controls were sera collected from two known highly positive local pigs (confirmed by dissection). The test results were determined by comparing the OD of each serum sample with a sample of negative serum samples (n = 8) at a probability level of p = 0.001 (Dorny et al., 2004b).

#### 2.1.4 Ethical statement

The study received approval of the Imperial College Research Ethics Committee, London and the Ethics Committee of the University of Kinshasa, the DRC (Reference: ESP/CE/008/2012). Further approval was obtained from the central Ministry of Health of the DRC, from the Provincial Ministry of Health of Bas-Congo province and from local district health and veterinary authorities. The research team was composed of human health professionals and veterinarians. The purpose of the study was explained to the community leaders of all villages and their permission requested before starting the study. Written consent was sought from each individual subject to take part in the study. For individuals younger than 16 years, permission was sought from their parents or guardians through written informed consent. All participants found positive for taeniasis were informed and provided with niclosamide (5 mg/kg) or praziquantel (40 mg/kg) depending on the arm of the clinical trial to which they belonged. Participating farmers were offered advice on the advantage of keeping pigs housed and improving the hygiene related to human open defecation. All farmers with positive pigs were advised on how to deal with the infected pork.

### 2.2 Statistical analysis

Both descriptive and analytical statistics were applied. Descriptive statistical analysis consisted in calculating frequencies and percentages of the variables of interest with their 95% confidence intervals (CI). A multivariate logistic regression was used to investigate the association between current taeniasis infection and individual (age, sex, use of latrine) as well as household (presence of latrine in the household and breeding pigs) variables recorded in questionnaires. Age variable was categorized using quartiles. To take into account the hierarchical structure of the data with individuals nested within households, a multilevel logistic regression model with two levels was applied. The modeling was done in three steps, and the following models were constructed:

Model 1 (‘empty model’), with no explanatory variables included;

Model 2, which included individual-level factors;

Model 3, with both individual and household-levels factors included.

The results of fixed effects (measure of association) were presented as odds ratios (OR) with 95% confident interval (CI). The results of random effects were presented as household random variance with standard errors (SE) and variance partition coefficient. Parameters were estimated using maximum likelihood estimation using adaptive quadrature in Stata 11 (Stata Corp Inc., TX, USA). The level of significance was set at 5%.

Spatial point patterns analysis was applied on the household geographical coordinates. For this, latitude and longitude coordinates were linked to the infection and questionnaire data collected at individual and household levels (multiple observations per location). In order to test whether extra cases of taeniasis tended to occur in proximity of other taeniasis cases, an exploratory analysis was conducted using the Ripley’s K function (Ripley, 1977). This function processes by dividing the average number of extra events (taeniasis infected individual) within a distance r of a randomly chosen event by the density of events in the area (Dixon, 2002). A file containing the coordinates of taeniasis positive participants’ households was read in R as Table and a grid was created using the maximum and minimum values for the x and y coordinates. An object of class “ppp” was then created on which the K test function was used. The confidence envelopes were calculated using 999 simulations under the Complete Spatial Randomness (CSR) hypothesis. The K function was then transformed into an L function which is easier to interpret visually (Besag, 1977). The same process was run for porcine cysticercosis cases. The analysis was done in R Studio version 0.98.1028 for Windows using the spatstat package.

A bivariate K function analysis (Dixon, 2002) was conducted to test the spatial dependence between the taeniasis positive human cases and cysticercosis infected pigs. This test measured the expected number of taeniasis positive human cases within a distance r from a cysticercosis sero-positive pig divided by the overall density of positive pigs. The analysis was done in R Studio version 0.98.1028 for Windows using the **splancs** package. Files were created and read as tables with the coordinates of the households of the positive human cases and the positive pigs. From these, point data sets were created. A polygon containing the points was generated from the datasets. The bivariate K function was calculated at 10 m increment distance bands using a vector of distances from 0 to 10 km. The choice of this distance was based on the recommendation of using distances shorter than one-half the shortest dimension of the study area (Dixon, 2002). Both axes of our study area were approximately 45 km. The results of the bivariate K function were represented in the form of an L function.

Spatial clusters were identified using the spatial scan statistic in the SaTScan software (Kulldorff, 1997). This test uses concentric circles or ellipses centered on each point with different sizes ranging from 0 to a predetermined limit. A likelihood ratio test is applied to each window, which is considered as a potential cluster. This enables to test the null hypothesis of absolute spatial randomness against the alternative hypothesis that there is an elevated risk within the window as compared to outside the window. In the present study, a circular window shape was used with a maximum spatial cluster size of 50% of the population at risk. The inference was done by using 999 Monte Carlo simulations (Kulldorff et al., 2007) where replications of the data set under the null hypothesis were generated. A Bernoulli distribution was used as the probability model and a cluster was significant if it had a maximum likelihood ratio higher than the maximum likelihood ratio from the most likely cluster generated in the random data set (Kulldorff, 1997, Kulldorff, 2010). The test was adjusted for the distribution of age categories. The level of significance was set at p = 0.05. All statistically significant clusters were mapped on to the study area using QGIS 2.

## 3 Results

### 3.1 Characteristics of the study population

Of a census population of 8139, a total of 5261 (64.6%) willing participants meeting inclusion criteria were enrolled in the study. This population belonged to 1699 households from 24 villages, with an average of three individuals sampled per household (ranging from 0 to 18). Of these, 4751 individuals (90.3%) provided a stool sample. After exclusion of individuals with incomplete files, a total of 4599 individuals were included for a full case analysis. Of these, 2240 (48.7%) were females and 2359 (51.3%) were males. The age ranged from 5 to 93 years with a median age of 23 years (IQR: 11-41). The age distribution was typical of rural areas in developing countries, with a majority from the younger age group. Among the included people, 1871 (40.7%) had no latrine in their households while 2211 (48%) did not use latrines; 605 (13.5%) were living in a household breeding pigs. A total of 338 pigs were sampled.

### 3.2 Proportion of taeniasis positivity and risk factors

Table 1 shows the proportion of positive human and pig samples in the different villages, grouped per health area. The overall proportion of taeniasis positivity was 23.4% (95%CI: 22.2-24.6) varying from 1 to 60% between villages. The overall proportion of positive pig samples was 45.6% (95% CI: 40.2-51) and varied from 0 to 94.1% between villages. Proportion of taeniasis positivity by age, sex and other explanatory variables are presented in Table 2. The highest positivity was determined in the 5-10 years age group (27.0% (95% CI: 24.4-29.7)). Logistic regression and multilevel analysis showed that individuals belonging to this age-group were significantly more likely to be infected than those in the 23-40 years (OR: 0.80 (95%CI: 0.66-0.97), p = 0.023) and 41-93 years (OR:0.62 (95%CI: 0.5-0.76), p < 0.001) age groups, but they were not different from the 11-22 years age-group (0.92 (0.76-1.1), p = 0.37). The empty model showed a significant variation in the likelihood of being infected between households (variance = 2.43 (SE = 0.29), p < 0.05). The value of the variance partition coefficient indicated that 42.48% of the variance could be attributed to differences between households. The variance remained significant and did not reduce after including individual nor household level factors in model 2 and 3. In fact, it increased from 2.43 (SE = 0.29) to 2.52 (SE = 0.30) and the variation partition coefficient increased as well from 42.48% to 43.39% in models 2 and 3.

### 3.3 Spatial point patterns analysis

**Fig. 2:**
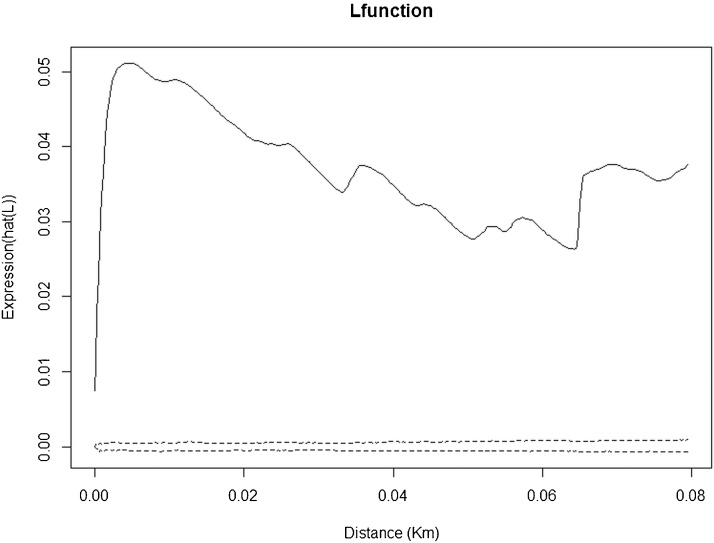
Ripley’s K function for taeniasis positive cases in the health zone of Kimpese. The black line represents the observed L function and the dashed lines represent the confidence envelopes calculated from 999 simulations under the hypothesis of complete spatial randomness (CSR). The observed L function lies above the envelopes, showing significant clustering.

**Fig. 3:**
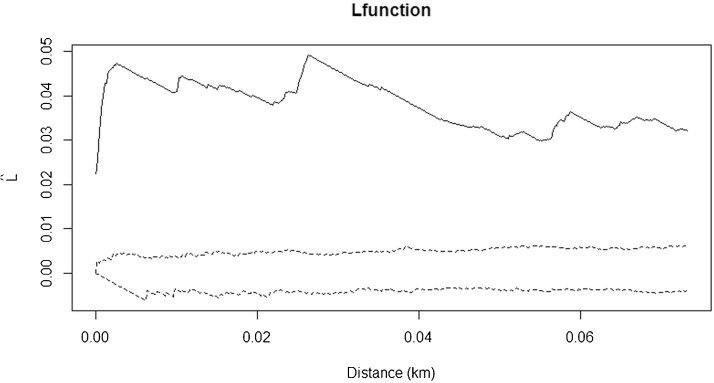
Ripley’s K function for porcine cysticercosis cases in the health zone of Kimpese. The black line represents the observed L function and the dashed lines represent the confidence envelopes calculated from 999 simulations under the hypothesis of complete spatial randomness (CSR). The observed L function lies above the envelopes, showing significant clustering.

**Fig. 4:**
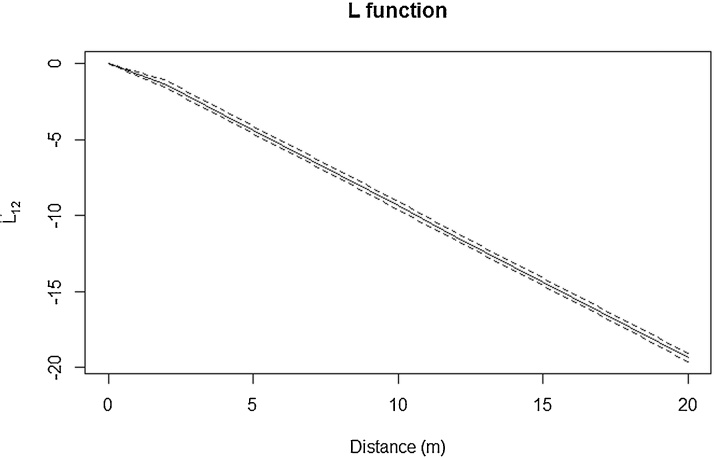
Bivariate K function for the copro-antigen positive human (taeniasis) and seropositive pigs (porcine cysticercosis). The dashed line represents the confidence envelopes calculated using 1000 simulations under the random toroidal shifts method and the black line represents the observed L function. The observed L function lies between the confidence envelopes, which means a complete spatial random distribution of both human and pigs infections.

**Fig. 5:**
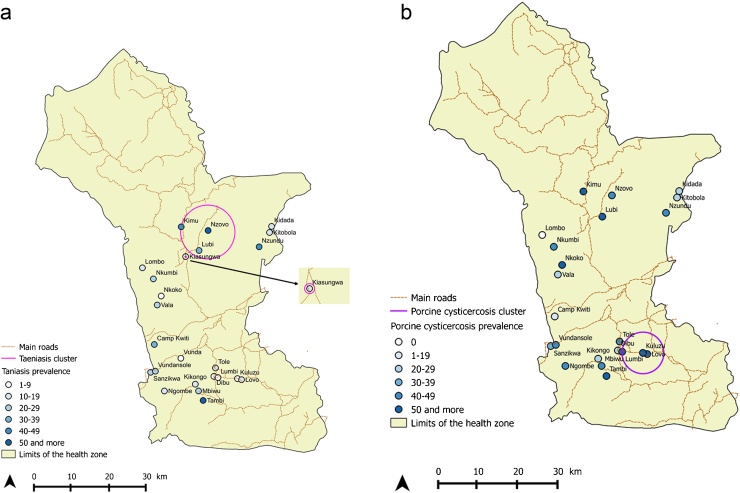
(a) Map of taeniasis positivity in villages of the rural health zone of Kimpese including location of taeniasis significant clusters. (b) Map of porcine cysticercosis positivity in villages of the rural health zone of Kimpese including location of porcine cysticercosis significant cluster.

Spatial point pattern analysis indicated significant spatial clustering of the taeniasis positive individuals at distance up to 80 m (Fig. 2), as do porcine cysticercosis cases (Fig. 3). Bivariate K function analysis showed that there was no significant spatial dependence of human taeniasis and porcine cysticercosis cases at any distance. Fig. 4 presents the most easily readable graph of this analysis, at 20 m scale. SatScan analysis revealed the presence of four significant clusters of human taeniasis in the study area. After adjusting for age, only two significant clusters were found. Both clusters were located in the health area of Kiasungwa, situated in the center of the Kimpese health zone. The main cluster partly covered two villages (Nzovo and Lubi); it included 276 cases and covered a radius of 5.74 km with a relative risk of 2.79 (p < 0.001). A secondary cluster was located in Kiasungwa village; it included 19 cases, with a radius of 0.063 km and a relative risk of 2.8 (p < 0.001). One cluster of porcine cysticercosis cases was found. The cluster was located in the health area of Lovo, situated in the south of the Kimpese health zone. It included 24 cases and covered a radius of 5.23 km straddling between three villages (Lovo, Kuluzu and Lumbi) with a relative risk of 1.95 (p = 0.019). There was no overlap between the human taeniasis clusters and the porcine cysticercosis cluster (Fig. 5).

## 4 Discussion

There is little available information on taeniasis/cysticercosis epidemiology in the DRC. The current study is the first step for upcoming control activities in the rural health zone of Kimpese where high human and porcine cysticercosis prevalence have been recently reported (Kanobana et al., 2011, Praet et al., 2010). The overall proportion of positivity in human was 23.4%, classifying this health zone as hyper-endemic. This high taeniasis positivity indicates a very high exposure risk to infective eggs. However, the infection displays a great heterogeneity as shown by the variability of the positivity (1% −60.7%) between villages and the significant between-household variance (variance = 2.43 (SE = 0.29), p < 0.05). Similar findings were also reported in two studies using coproantigen detection in Peru (9 villages) (Garcia et al., 2003a) and Guatemala (4 villages) (Allan et al., 1996). These studies reported significant household clustering and taeniasis prevalence ranges of 0–6.7% (overall prevalence = 2.8%) and 0–5.1% (overall prevalence = 2.7%) between villages respectively. Differences in consumption of infected pig meat or household dietary and cooking habits could explain such heterogeneity of taeniasis infection. Further studies are warranted in our study area to explore influence of those factors on the observed heterogeneity. The taeniasis positivity in each village of our study area was much higher than a prevalence of 0.3% found previously, using coprology, in one selected village of the same health zone (Kanobana et al., 2011). This difference can be partially explained by the sensitivity of copro-antigen ELISA, which is 2-10 times higher than coprology (Allan et al., 1990) or by other factors underlying the aforementioned heterogeneous trend of taeniasis in endemic areas. However, these high proportion of positive humans and pigs must be cautiously interpreted. Indeed, the copro-Ag ELISA is only genus specific and as such cannot differentiate between *T. solium* and **Taenia* saginata* infections (Allan et al., 2003), while Ag-ELISA cannot distinguish *T. solium* from *Taenia hydatigena* infections in pig (Dorny et al., 2004a). It is therefore probable that the proportions of positive human and pigs could be overestimated. Nonetheless, a Bayesian modeling estimating the test characteristics of coprology, coproantigen ELISA and PCR for the diagnosis of taeniasis, yielded estimates of 84.5% sensitivity and 92% specificity for the ELISA coproantigen, based on samples from Zambia, which is close to DRC (Praet et al., 2013). Moreover, cattle farming is not practiced in the region of Kimpese (Gutu Kia Zimi, 2014). Likewise there is a very limited consumption of beef in the study area (personal observation), both factors indicating low risk for *T. saginata*. Regarding **Taenia* asiatica*, we believe that its presence and continued transmission is not likely to occur in our study area, due to its known geographical distribution, mainly in some Asian countries (Ale et al., 2014) and the unlikely migration from Asia to these remote villages. Regarding *T. hydatigena*, only one case has been reported in the Eastern province of the DRC (Chartier et al., 1990). Also *T. hydatigena* prevalence in sub-saharian African countries seems to be low. Reported prevalence were 2.2% in Nigeria (Fabiyi, 1979), 6.7% in Ghana (Permin et al., 1999), 1.4% and 6.6% in Tanzania (Ngowi et al., 2004, Braae et al., 2015), 6.1% in Zambia (Dorny et al., 2004b) and few cases in South Africa (Horak, 1980). Based on these data, we do not expect high number of *T. saginata* and *T. hydatigena* cases and at the very most only slight overestimates for both human and pig infections could be expected.

Compared with similar studies (using copro-Ag-ELISA), the overall taeniasis positivity determined in this study is higher than prevalences of 6.3% and 11.9% found in Zambia (Mwape et al., 2012, Mwape et al., 2013); 5.2% found in Tanzania (Mwanjali et al., 2013) and the range of 2.8%–14% reported in Mexico and Peru (Garcia et al., 2003a, Garcia-Noval et al., 1996, Sarti et al., 1992). In the absence of data on pig meat production, consumption and cooking habits, the high proportion of taeniasis found here can be explained by the high proportion of sero-positive pigs which in turn can be explained by low hygiene standards, free management of pigs and pig trade system practiced in our study area. Indeed, 40.7% of the study population had no latrine in their households, 48% did not always use latrines and all pigs were reared on free range. We found an 45.6% overall proportion of sero-positive pigs, in the range of reported by Praet et al. (2010) in five selected villages of the same area (41.2%; CI95%: 33-49). Moreover, these authors reported that in the pig trade chain from villages of this area to the capital town, highly infected animals were excluded by pig farmers and/or buyers through tongue inspection and kept in the villages. This primarily explains the high porcine cysticercosis but it is also plausible that these infected pigs kept for local consumption in a context of lack of meat inspection might increase the risk of taeniasis among village communities.

A tapeworm carrier is the central node of cysticercosis transmission since he/she might transmit this infection to himself/herself (through autoinfection), to other community members and to pigs. It was previously shown that frequency of taeniasis in patients with neurocysticercosis may reach 15%, and patients with more cerebral cysticerci have a higher probability of carrying concomitantly a tapeworm (Garcia and Del Brutto, 1999, Gilman et al., 2000). The high taeniasis positivity found here may thus imply high risk for human (neuro) cysticercosis in our study area, which is in line with 21.6% of human cysticercosis prevalence reported previously in one selected village (Kanobana et al., 2011).

A remarkable finding of this study is that age appears to be significantly associated with taeniasis. The highest positivity was found in subjects belonging to the 5-10 years age group. This finding is in line with the two aforementioned studies conducted in Peru and Guatemala (n = 1620 and 3399) (Allan et al., 1996, Garcia et al., 2003a) but not with comparable epidemiologic studies performed in Tanzania and Zambia (n = 718 and 830) (Mwanjali et al., 2013, Mwape et al., 2012). The scarcity of such age-specific infection pattern probably reflects the paucity of data, due to a low number of tapeworm carriers found in studies with small sample sizes. In Peru, Garcia et al. reported significantly higher prevalence in individuals of 10 years old or younger (23/595, OR 1.83, p = 0.042) among 1620 post treatment tested samples (Garcia et al., 2003a). In Guatemala, children in 5–10 years age group were significantly more likely to be infected than in 0-4 years age group (RR = 1.43; CI = 1.12-1.83; p < 0.04), though the highest prevalence was found in the 30-39 years age group (Allan et al., 1996). Despite the exclusion of under 5 years, the age-related proportion of taeniasis positivity found in the present study is suggestive of young age of infection. Currently, it is unclear whether this age relation in taeniasis is due to meat consumption behavior or rather the consequence of (immune-) protection against the tapeworm development in older individuals. Further studies, including other age-related factors not assessed in this study, are needed to establish the consistence and the implications of these findings in control activities design. If the 5-10 years age group (approximately primary school age) is indeed the most affected, then inclusion of taeniasis in the combined school-based epidemiological surveys for schistosomiasis and soil-transmitted helminth infections (WHO, 2004, WHO, 2011) could be recommended in co-endemic areas. However, as all age-groups are affected by taeniasis, MDA against taeniasis targeting the entire population as recommended by WHO (WHO, 2012) remains probably the most advisable control strategy for this study area at this point.

As in a number of other studies, geo-spatial analysis conducted in our study revealed significant clustering of taeniasis (Mwape et al., 2012, Raghava et al., 2010, Rodriguez-Canul et al., 1999) and porcine cysticercosis (Diaz et al., 1992, Mwape et al., 2012, Ngowi et al., 2010, Widdowson et al., 2000). This is indicative of the spatial heterogeneity of *T. solium* infections in endemic areas. In our study taeniasis spatial heterogeneity was partly due to the age distribution of human host, as revealed by adjusted satscan analysis. However, no geospatial correlation of cysticercosis infected pigs and human positive taeniasis was found. Similar lack of spatial correlation was reported by Morales et al. (2008) in Mexico and explained by the combination of roaming behavior of pigs that covered large distances (2-5 km) daily (Copado et al., 2004) and mobile patterns of human in the study area. A study conducted in Kenya, geographically closer to our study area, found that free ranging pigs traveled an average of 4,340 m in a 12 h period and spent on average 47% of their time outside their homestead of origin and on average only 1.3% of their time interacting with the latrine area in their homestead of origin (Thomas et al., 2013). In such situation, ingestion of infective *T. solium* eggs by a free-roaming pig could therefore occur anywhere far from the homestead of origin and in the immediate circle of any tapeworm carrier. It could be that such situation occurs in our study area where all pigs are free roaming and human, essentially farmers, move around for agricultural work. Also, pigs slaughtered in the villages are not consumed only in the immediate vicinity of breeders, but also by members of other households in the same village or in neighboring villages, spreading randomly the geographic risk of taeniasis. Hence, the geographical proximity of a human with an infected pig does not determine the risk of being taeniasis infected in our study area and vice versa. These results, however, are opposed to what was reported in studies by Lescano et al. (Lescano et al., 2007) and O’Neal et al. (O'Neal et al., 2012) in Peru, where swine sero-positive pigs clustered near tapeworm carriers (distance < 500m) and taeniasis clustered within 100 m of a tongue positive pig respectively. This discrepancy could be explained by differences in several factors (cultural, geographical, human migration, pig-rearing style, etc.) that may influence the geographic distribution of *T. solium* infections. These conflicting findings call for the need for context-specific situational analysis.

The small pig sample size and the duration of sampling should be taken in account as limiting factors when interpreting these results. Indeed, an outbreak of African swine fever occurred in April 2011 and reduced the pig population. New pigs were imported and could therefore not be linked to infection by local tapeworm carriers. Also, in some cases pig sampling could not be carried out simultaneously with that of humans due to reluctance of some farmers. This might have weakened the correlation, in space and time, between taeniasis cases and porcine cysticercosis cases. Nevertheless, our findings imply that the distribution of infected pigs alone cannot be used as an indicator to determine where screening and treatments should be targeted in our study area. In addition, despite the identification of hotspots for both taeniasis and cysticercosis, their widespread distribution in this endemic area suggest that control measures must be applied indiscriminately to all communities of our study area, with a particular attention to children under ten years of age.

## 5 Conclusion

This study provides, for the first time, important information on taeniasis in a hyper endemic rural area of the DRC. Data highlight the role of age in the heterogeneous pattern of taeniasis, the absence of spatial correlation between taeniasis and porcine cysticercosis and the need of control measures in this endemic area. The highest taeniasis positivity in the 5-10 years age group is suggestive of including taeniasis in the school-based epidemiological surveys of schistosomiasis and soil-transmitted helminth infections. Further studies are needed to better understand this age pattern and the heterogeneous distribution of *T. solium* infections in this area. These data can contribute to the design of appropriate control for *T. solium* in the DRC and potential for integrated control strategies with other NTDs.

## Figures and Tables

**Table 1:** Prevalences of taeniasis and porcine cysticercosis in different villages of the study area.

Health area	Village	Positive in Copro-Ag ELISA (taeniasis)		Seropositive in Ag-ELISA (porcine cysticercosis)	
		Proportion	% (95%CI)	Proportion	% (95%CI)
All	All	1112/4751	23.4 (22.2–24.6)	154/338	45.6 (40.2–51.0)
Kiasungwa	Kiasungwa	170/899	18.9 (16.4–21.7)	–	–
	Nzovo	153/252	60.7 (54.4–66.8)	4/9	44.4 (13.7–78.8)
	Kimu	82/171	48.0 (40.3–55.7)	9/14	64.3 (35.1–87.2)
	Lubi	37/96	38.5 (28.8–49.0)	4/6	66.7 (22.3–95.7)

Malanga	Nkumbi	27/132	20.5 (13.9–28.3)	8/18	44.4 (21.5–69.2)
	Vala	15/73	20.5 (12.0–31.6)	1/5	20 (0.5–71.6)
	Nkoko	2/26	7.7 (0.9–25.1)	3/5	60 (14.7–94.7)
	Lombo	13/89	14.6 (8.0–23.7)	0/7	0 (0.0–41.0)

Kilueka	Kitobola	78/416	18.8 (15.2–22.9)	6/26	23.1 (9.0–43.6)
	Nzundu	87/210	41.4 (34.7–48.4)	12/21	47.6 (25.7–70.2)
	Kidada	21/123	17.1 (10.9–24.9)	9/31	29 (14.2–48)

Lovo	Lovo	31/181	17.1 (11.9–23.4)	12/17	70.6 (44.0–89.7)
	Kuluzu	3/72	4.2 (0.9–11.7)	16/17	94.1 (71.3–99)
	Kikongo	27/148	18.2 (12.4–25.4)	4/17	23.5 (6.8–49.9)
	Tambi	70/127	55.1 (46–63.9)	12/21	57.1 (34.0–78.2)
	Lumbi	16/256	6.3 (3.6–10)	10/18	55.6 (30.8–78.5)
	Mbiwu	45/169	26.6 (20.1–34)	18/28	44.4 (21.5–69.2)
	Tole	22/124	19.4 (12.8–27.4)	2/5	40 (5.3–85.3)
	Dibu	1/101	1 (0.0–5.4)	2/9	22.2 (2.8–60.0)

Viaza	Camp Kwiti	10/32	31.3 (16.7–50.0)	1/10	10 (0.3–44.5)
	Vunda	7/80	8.8 (3.6–17.2)	–	–

Vundansole	Vundansole	71/340	20.9 (16.8–25.7)	11/26	42.3 (23.4–63.1)
	Sanzikwa	77/369	20.9 (16.9–25.4)	3/8	37.5 (8.5–75.5)
	Ngombe	45/265	17.0 (12.7–22.1)	9/20	45.0 (23.1–68.5)

**Table 2:** Patterns of taeniasis by explanatory variables and multilevel analysis odds ratio (OR).

Characrteristics	Positive in Copro-Ag-ELISA (taeniasis)		Multilevel analysis	
	Proportion	% (95%CI)	OR (95%CI)	p
All	1075/4599	23.4 (22.2–24.6)	–	–

Sex				
F	542/2359	23.0 (21.3–24.7)	1	
M	533/2240	23.8 (22.1–25.6)	1.05 (0.9–1.2)	0.5

Age categories				
5-10 years	300/1113	27.0 (24.4–29.7)	1	
11-22 years	289/1143	25.3 (22.8–27.9)	0.92 (0.76–1.1)	0.37
23-40 years	267/1169	22.8 (20.5–25.4)	0.80 (0.66–0.97)	**0.023**
41-93 years	219/1174	18.6 (16.5–21.0)	0.62 (0.5–0.76)	**<0.001**

Presence of a latrine				
No	458/1871	24.5 (22.6–26.5)	1	
Yes	617/2728	22.6 (21.1–24.2)	0.9 (0.78–1.17)	0.14

Use of the latrine				
No	537/2211	24.3 (22.5–26.1)	1	
Yes	538/2388	22.5 (20.9–24.3)	0.9 (0.79–1.04)	0.15

Breeding pigs^a^				
No	938/3994	23.5 (22.2–24.8)	1	
Yes	137/605	22.6 (19.4–26.2)	0.95 (0.78–1.17)	0.65

a All pigs were reared at free-range.
